# Supplementary material for: Total daily physical activity, brain pathologies, and parkinsonism in older adults
Source: PLoS One. 2020 Apr 29;15(4):e0232404. doi: 10.1371/journal.pone.0232404 (PMC7190120; doi:10.1371/journal.pone.0232404)
Supplement: S1 Table — (DOCX) [file pone.0232404.s001.docx]

**Supplementary Table e-1**. Comparison of participants included and excluded from these analyses due to missing clinical data.

| **Covariates** | **Included (n=447)**  **Mean (SD) or N (%)** | **Excluded (n=72)**  **Mean (SD) or N (%)** | **P-value^*^** |
| --- | --- | --- | --- |
| **Demographic** |  |  |  |
| Age at death (years) | 90.9 (6.2) | 89.6 (6.2) | 0.113 |
| Age at the actigraphic recording | 88.8 (6.2) | 87.4 (6.2) | 0.107 |
| Female | 316 (71) | 62 (86) | 0.006 |
| Years of education | 14.7 (2.9) | 14.3 (2.6) | 0.399 |
| Marital status  Never married  Married  Widowed  Divorced  Separated | 34(8)  97(23)  261(62)  26(6)  0 | 5(9)  11(19)  38 (67)  3 (5)  0 | 0.901 |
| **Clinical** |  |  |  |
| Sum of history of vascular risk factors | 1.3 (0.8) | 1.3 (0.8) | 0.679 |
| Hypertension | 315 (70) | 53 (74) | 0.586 |
| Diabetes Mellitus | 97 (22) | 15 (21) | 0.868 |
| Smoking (ever in life) | 176 (40) | 27 (38) | 0.741 |
| Sum of vascular diseases | 0.8 (0.9) | 0.7 (0.9) | 0.685 |
| Stroke | 95 (22) | 14 (19) | 0.680 |
| Heart attack | 87 (19) | 14 (19) | 0.997 |
| Heart failure | 57 (15) | 12 (18) | 0.520 |
| Lower extremities claudication | 121 (27) | 21 (29) | 0.719 |
| Neuroleptic medication use | 86 (19) | 6 (8) | 0.037 |
| **Quantitative metrics extracted from multi-day recordings** |  |  |  |
| Total daily physical activity (activity counts/day) | 1.44 × 10^5^ (1.13× 10^5^) | 1.66 × 10^5^ (1.47 × 10^5^) | 0.591 |
| Intensity of daily physical activity (activity counts/active hours) | 0.18× 10^5^ (0.09× 10^5^) | 0.20 × 10^5^ (0.10 × 10^5^) | 0.681 |
| K_RA_(probability metric of sleep disruption due to movement)^**^ | 0.028 (0.008) | 0.027 (0.010) | 0.234 |

^*^We employed t-test to compare continuous covariates included in this table, chi square to compare categorical variables, and Wilcoxon rank test to compare quantitative actigraphic metrics and vascular risk factor and diseases summary measures.

^**^K_RA_ was available in 378 of included and 59 of excluded participants.
