# Supplementary material for: Total daily physical activity, brain pathologies, and parkinsonism in older adults
Source: PLoS One. 2020 Apr 29;15(4):e0232404. doi: 10.1371/journal.pone.0232404 (PMC7190120; doi:10.1371/journal.pone.0232404)
Supplement: S2 Table — (DOCX) [file pone.0232404.s002.docx]

**Supplementary Table e-2.** Association of total daily physical activity proximate to death with postmortem brain pathology indices.^*^

| **Outcome** | **Estimate**  (β (SE) or OR (95% CI) | **p-Value** |
| --- | --- | --- |
| **AD pathology, β (SE)** | 0.020 (0.016) | 0.210 |
| **Lewy body pathology, OR (95% CI)** | 0.88 (0.73 – 1.07) | 0.206 |
| **Nigral neuronal loss, OR (95% CI)** | **0.66 (0.46 – 0.93)** | **0.018** |
| **TDP-43, OR (95% CI)** | 1.14 (0.96 – 1.36) | 0.147 |
| **Hippocampal sclerosis, OR (95% CI)** | 0.93 (0.70 – 1.24) | 0.625 |
| **Macroinfarcts, OR (95% CI)** | **0.81 (0.67 – 0.97)** | **0.021** |
| **Microinfarcts, OR (95% CI)** | 0.91 (0.75 – 1.09) | 0.289 |
| **Arteriolosclerosis, OR (95% CI)** | 0.96 (0.80 – 1.16) | 0.673 |
| **Atherosclerosis, OR (95% CI)** | 0.94 (0.77 – 1.14) | 0.526 |
| **Cerebral Amyloid Angiopathy, OR (95% CI)** | 1.09 (0.92 – 1.30) | 0.327 |

^*^Each row in the table shows the results of a separate model which used either a linear or logistic regression to examine the association between total daily physical activity and a different brain pathology as shown in the left column. All models controlled for age at death and sex. Estimates illustrate that a higher level of total daily physical activity is associated with a lower odds of macroinfarcts and nigral neuronal loss.
