# Supplementary material for: Total daily physical activity, brain pathologies, and parkinsonism in older adults
Source: PLoS One. 2020 Apr 29;15(4):e0232404. doi: 10.1371/journal.pone.0232404 (PMC7190120; doi:10.1371/journal.pone.0232404)
Supplement: S3 Table — (DOCX) [file pone.0232404.s003.docx]

**Supplementary Table e-3.** Association of total daily physical activity and indices of brain pathologies with parkinsonism proximate to death controlling for potential confounders.^*^

| **Primary Predictor** | **Estimate**  β (SE) | **p-Value** |
| --- | --- | --- |
| **Total daily physical activity ^A^** | -0.331 (0.051) | <0.001 |
| **Total daily physical activity ^A*^** | -0.296 (0.051) | <0.001 |
| **Total daily physical activity ^B^** | -0.304 (0.052) | <0.001 |
| **Total daily physical activity ^B*^** | -0.277 (0.052) | <0.001 |
| **Total daily physical activity ^C^** | -0.337 (0.051) | <0.001 |
| **Total daily physical activity ^C*^** | -0.304 (0.051) | <0.001 |
| **Total daily physical activity ^D^** | -0.323 (0.051) | <0.001 |
| **Total daily physical activity ^D*^** | -0.292 (0.051) | <0.001 |
| **Total daily physical activity ^E^** | -0.288 (0.050) | <0.001 |
| **Total daily physical activity ^E*^** | -0.257 (0.050) | <0.001 |

^*^Each row in the table shows the results for the term total daily physical activity from a separate linear regression model which examined the association between total daily physical activity and parkinsonism controlling for a different potential confounder. Confounders included: **A**: Education, **B**: Marital Status, **C**: Vascular risk factors, **D**: Vascular Disease, **E**: Use of neuroleptics. The initial model (**letter without star**) in each row included a term for total daily physical activity and controlled for a different confounder as well as age at death, sex. The second model (letter with star) in each row added terms for brain pathologies to the terms included in the initial model. Controlling for these confounders did not change the conclusion that a higher level of total daily physical activity was associated with less parkinsonism severity independent of indices of brain pathology.
