# Supplementary material for: Total daily physical activity, brain pathologies, and parkinsonism in older adults
Source: PLoS One. 2020 Apr 29;15(4):e0232404. doi: 10.1371/journal.pone.0232404 (PMC7190120; doi:10.1371/journal.pone.0232404)
Supplement: S4 Table — (DOCX) [file pone.0232404.s004.docx]

**Supplementary Table e-4.** Association of total daily physical activity and indices of brain pathologies with parkinsonism proximate to death after exclusion of participants with a clinical diagnosis of Parkinson disease (PD) or those receiving neuroleptic medications.

| **Model Terms** | **Participants without PD diagnosis**  **(n=432)** | | **Participants not taking neuroleptics**  **(n=361)** | |
| --- | --- | --- | --- | --- |
|  | **Model 1-TDPA**  Est. (SE),p-value | **Model 3-TDPA+Path**  Est. (SE),p-value | **Model 1-TDPA**  Est. (SE),p-value | **Model 3-TDPA+Path**  Est. (SE),p-value |
| **Total daily physical activity** | **-0.294 (0.052)**  **<0.001** | **-0.270 (0.052)**  **<0.001** | **-0.224 (0.056)**  **<0.001** | **-0.203 (0.056)**  **<0.001** |
| **AD pathology** |  | -0.115 (0.103)  0.266 |  | -0.161 (0.115)  0.161 |
| **Lewy body pathology** |  | 0.026 (0.140)  0.853 |  | 0.006 (0.147)  0.968 |
| **Nigral neuronal loss** |  | 0.321 (0.213)  0.133 |  | 0.179 (0.224)  0.424 |
| **TDP-43** |  | -0.042 (0.132)  0.752 |  | 0.010 (0.138)  0.945 |
| **Hippocampal sclerosis** |  | 0.228 (0.199)  0.254 |  | 0.151 (0.216)  0.486 |
| **Macroinfarcts** |  | 0.165 (0.123)  0.183 |  | 0.282 (0.129)  0.030 |
| **Microinfarcts** |  | 0.009 (0.125)  0.941 |  | 0.045 (0.132)  0.735 |
| **Arteriolosclerosis** |  | 0.191 (0.130)  0.142 |  | 0.203 (0.138)  0.142 |
| **Atherosclerosis** |  | 0.393 (0.136)  0.004 |  | 0.276 (0.146)  0.059 |
| **Cerebral Amyloid Angiopathy** |  | -0.015 (0.128)  0.907 |  | 0.010 (0.136)  0.943 |

^*^Each column shows the results of a separate linear regression model showing the association of total daily physical activity with global parkinsonism proximate to death with and without indices of brain pathology after exclusion of either participants with a clinical diagnosis of PD or those who were taking neuroleptic medications which can cause increased severity of parkinsonian signs. All models controlled for age at death and sex. The association of total daily physical activity and parkinsonism is unchanged is not attenuated by the addition of terms for brain pathologies.
