# Supplementary material for: Total daily physical activity, brain pathologies, and parkinsonism in older adults
Source: PLoS One. 2020 Apr 29;15(4):e0232404. doi: 10.1371/journal.pone.0232404 (PMC7190120; doi:10.1371/journal.pone.0232404)
Supplement: S5 Table — (DOCX) [file pone.0232404.s005.docx]

**Supplementary Table e-5.** Association of total daily physical activity and indices of brain pathologies with parkinsonism proximate to death controlling for k_RA_, probability metric of sleep disruption due to movement).*****

| **Model Terms** | **Model 1**  Estimate (Standard Error), p-value | **Model 2**  Estimate (Standard Error), p-value | **Model 3**  Estimate (Standard Error), p-value |
| --- | --- | --- | --- |
| **Total daily physical activity (TDPA)** | **-0.370 (0.053)**  **<0.001** |  | **-0.337 (0.054)**  **<0.001** |
| **K_RA_** | 33.982 (7.339)  <0.001 |  | 31.715 (7.436)  <0.001 |
| **AD pathology** |  | -0.136 (0.110)  0.219 | -0.163 (0.104)  0.117 |
| **Lewy body pathology** |  | -0.068 (0.150)  0.650 | -0.085 (0.141)  0.546 |
| **Nigral neuronal loss** |  | 0.602 (0.212)  0.005 | 0.331 (0.203)  0.104 |
| **TDP-43** |  | -0.056 (0.143)  0.697 | 0.050 (0.136)  0.712 |
| **Hippocampal sclerosis** |  | 0.228 (0.219)  0.299 | 0.098 (0.207)  0.637 |
| **Macroinfarcts** |  | 0.286 (0.133)  0.032 | 0.219 (0.125)  0.082 |
| **Microinfarcts** |  | 0.121 (0.134)  0.368 | 0.107 (0.126)  0.397 |
| **Arteriolosclerosis** |  | 0.209 (0.138)  0.132 | 0.164 (0.130)  0.209 |
| **Atherosclerosis** |  | 0.271 (0.150)  0.072 | 0.267 (0.141)  0.059 |
| **Cerebral Amyloid Angiopathy** |  | 0.041 (0.135)  0.759 | 0.081 (0.127)  0.526 |

*Each column shows the results of a separate linear regression model showing the association of the predictors listed in the left hand column. All models controlled for age at death and sex (not shown). Total daily physical activity remains independently associated with global parkinsonism when controlling for **K_RA_**, sleep related movement and indices of brain pathology.
