# Supplementary material for: Total daily physical activity, brain pathologies, and parkinsonism in older adults
Source: PLoS One. 2020 Apr 29;15(4):e0232404. doi: 10.1371/journal.pone.0232404 (PMC7190120; doi:10.1371/journal.pone.0232404)
Supplement: S6 Table — (DOCX) [file pone.0232404.s006.docx]

**Supplementary Table e-6**. Distribution of indices of Alzheimer’s disease and related disorders (ADRD) pathologies in the Memory and Aging Project compared to 2 other community-based longitudinal clinical-pathological studies of aging(1).

| **Postmortem Indices** | **Memory and Aging Project** | **Baltimore Longitudinal Study of Aging**^(1)^ | **90+ Study**^(1)^ |
| --- | --- | --- | --- |
| NIA-Reagan AD pathological diagnosis | 69% | 56% | 67% |
| Lewy body pathology | 28% | 6% | 6% |
| TDP-43 | 36% |  | 31% |
| Hippocampal sclerosis | 11% |  | 29% |
| Macroinfarcts | 39% | 44% | 30%^(2)^ |
| Microinfarcts | 32% |  |  |

References:

1. Rahimi J, Kovacs GG. Prevalence of mixed pathologies in the aging brain. Alzheimers Res Ther. 2014 Dec;6(9):82.

2. M. Corrada M, J. Berlau D, H. Kawas C. A Population-Based Clinicopathological Study in the Oldest-Old: The 90+ Study. Curr Alzheimer Res. 2012 Jun 1;9(6):709–17.
